# Synthesizing Multiple Stakeholder Perspectives on Using Virtual Reality to Improve the Periprocedural Experience in Children and Adolescents: Survey Study

**DOI:** 10.2196/19752

**Published:** 2020-07-17

**Authors:** Naseem Ahmadpour, Andrew David Weatherall, Minal Menezes, Soojeong Yoo, Hanyang Hong, Gail Wong

**Affiliations:** 1 Design Lab School of Architecture, Design and Planning The University of Sydney Darlington, NSW Australia; 2 Division of Child and Adolescent Health The University of Sydney Sydney Australia; 3 Department of Anaesthesia The Children's Hospital at Westmead Westmead Australia; 4 Sydney Medical School The University of Sydney Sydney Australia

**Keywords:** virtual reality, periprocedural anxiety, children, adolescents, stakeholder perspective, design, VR, pediatrics, patient experience, app, eHealth

## Abstract

**Background:**

Virtual reality (VR) technology is a powerful tool for augmenting patient experience in pediatric settings. Incorporating the needs and values of stakeholders in the design of VR apps in health care can contribute to better outcomes and meaningful experiences for patients.

**Objective:**

We used a multiperspective approach to investigate how VR apps can be designed to improve the periprocedural experiences of children and adolescents, particularly those with severe anxiety.

**Methods:**

This study included a focus group (n=4) and a survey (n=56) of clinicians. Semistructured interviews were conducted with children and adolescents in an immunization clinic (n=3) and perioperative setting (n=65) and with parents and carers in an immunization clinic (n=3) and perioperative setting (n=35).

**Results:**

Qualitative data were examined to determine the experience and psychological needs and intervention and design strategies that may contribute to better experiences for children in three age groups (4-7, 8-11, and 12-17 years). Quantitative data were used to identify areas of priority for future VR interventions.

**Conclusions:**

We propose a set of ten design considerations for the creation of future VR experiences for pediatric patients. Enhancing patient experience may be achieved by combining multiple VR solutions through a holistic approach considering the roles of clinicians and carers and the temporality of the patient’s experience. These situations require personalized solutions to fulfill the needs of pediatric patients before and during the medical procedure. In particular, communication should be placed at the center of preprocedure solutions, while emotional goals can be embedded into a procedure-focused VR app to help patients shift their focus in a meaningful way to build skills to manage their anxiety.

## Introduction

A fundamental role of clinicians looking after children having medical procedures in hospitals is to try to minimize any distress associated with that procedure. A negative experience of a medical procedure has implications more extensive than the immediate distress of that event. The imprint will be left on the child for all subsequent medical procedures. A range of approaches may be attempted as a periprocedural intervention (ie, intervention prior, during, or after the medical procedure) to mitigate such negative experiences, especially in the context of significant anxiety associated with needles in children requiring vaccinations [1]. In-hospital sedation services facilitate vaccination in patients who are otherwise unable to complete their immunization schedule due to severe avoidance behavior. Improved immunization experiences for patients, their caretakers, and staff may reduce the need for pharmacological intervention in the form of sedation, which may be difficult to administer or sometimes harmful [2]. Other approaches, such as exposure therapy, require long-term time commitments and significant resources [3]. In this paper, we present an exploratory study into ways through which virtual reality (VR) might best be employed to enhance the pediatric patient experience.

Technology-mediated solutions for focus-shifting or distraction positively alter the periprocedural experiences of children, to reduce anxiety and decrease pain response [4]. Current evidence suggests efficacy for technologies such as hand-held video games [5], tablet computers [6], immersive 360 videos [7], and interactive VR games [8]. VR technology has been explicitly explored as an anxiety management intervention in the context of vaccination [9] and acute procedures such as laceration repair and wound care [10]. With recent advances in commercial VR Head-Mounted Display (HMD) devices (eg, Oculus Quest by Oculus VR), the availability of this technology has dramatically increased. Recent market research [11,12] found that of 1917 children (aged <15 years) surveyed in the United States of America, only 19% were unaware of VR technology in the Spring of 2017. This mainstream awareness of VR technology presents an opportunity that we can leverage to develop effective interventions to support pediatric treatment.

The efficacy of VR apps is often attributed to the distraction created by the immersive environment, which creates an illusion of presence in the virtual world [13] and therefore reduces the user’s cognitive resources to attend to distressing stimuli. Distraction in VR may be achieved in two ways: passive and active [14]. Passive engagement can be in the form of viewing an immersive 360-degree video without guiding the user’s attention. Active distraction engages the user in gameplay or cognitive tasks in order to shift their focus to virtual objects and away from the negative stimuli [15]. Distraction can be more effective if patients are engaged in emotionally relevant VR experiences [16] as it enables them to dissociate their virtual and physical bodies [17].

Current examples of VR for managing pediatric anxiety do not exploit the multitude of strategies possible in VR, particularly active distraction. Additionally, there is a lack of knowledge about the needs of pediatric patients as well as the role of other stakeholders involved in patient care, including clinicians and parents, that can be incorporated into future pediatric VR solutions [18]. This study aimed to investigate opportunities for designing VR apps for children and adolescents in order to improve their experience and help them manage anxiety in the context of perioperative care and immunization.

To achieve this aim, we followed a unique multiperspective and qualitative approach. We collected in-depth data from multiple stakeholders to explore factors that can inform the design of future VR solutions. We conducted a focus group and surveyed clinicians to collect their views on typical strategies for managing pediatric anxiety in periprocedural settings. To understand patient responses to current anxiety management strategies, we conducted semistructured interviews with children and their carers.

## Methods

This study included a focus group and survey of clinicians, in addition to interviews with children and parents using protocols approved by the Sydney Children’s Hospitals Network Human Research Ethics Committee (HREC Reference Number–LNR/18/SCHN/160 HREC/17/SCHN/429). All study participants were recruited from The Children’s Hospital at Westmead, NSW in Australia and provided written informed consent before participation. Focus groups and semistructured interview sessions were audio-recorded.

### Clinicians

#### Focus Group

Four health professionals were recruited to participate in a 90-minute focus group convened by two researchers; one anesthetist and one human-computer interaction (HCI) researcher. The aim was to gain a better understanding of the strategies used by clinicians to manage periprocedural anxiety in their pediatric patients. The session started with a brief explanation of VR technology, followed by questions that guided the discussion, like “Do you use any particular strategies to shift the focus of children during the procedures?” and “How do you choose which approach will work for which patient?”

#### Survey

A total of 56 clinicians were recruited by email and direct approach to complete a survey, repeated three times, and focusing on children in three age groups: 4-7, 8-11, and 12-17 years old. The questionnaire was developed by researchers with a background in clinical anesthesia based on their experience. Participants rated the likelihood of 13 items to induce anxiety in children and adolescents in the preoperative, operative, and postoperative phases (5-point scales, ranging from “not at all” to “very much”). Participants were then asked to describe strategies based on their experience that made the patient feel good, calm, or reassured before going in for an operation.

### Children and Adolescents

#### Semistructured Interview in the Immunization Clinic

Three participants were recruited from the immunization clinic for 90-minute interview sessions convened by two HCI researchers and a child-life therapist. Participants attended the meetings with their parent(s) but were interviewed without them being present in the room. The interview started with, “Can you tell us about what it was like last time you came in for immunizations?” We then explained the VR technology and asked, “what would you like to see or do in VR?” Drawing and clay play were used to help participants communicate their ideas.

#### Semistructured Interviews in the Perioperative Setting

Patients presenting for day surgery were approached in the hospital preoperative waiting room for 15-minute interview sessions. Members of the research team with a background in clinical anesthesia developed questions for semistructured interviews that included a series of rating scales to capture how much the participant knew about the hospital visit on the day of the procedure (4-point scale, ranging from “nothing at all” to “everything”). Additional open-ended questions inquired about things that make children feel good about going to sleep for an operation and things about which they were concerned. A final question asked about the quality of previous experiences of anesthesia where relevant (4-point scale, ranging from “very bad” to “very good”).

### Parents and Carers

#### Semistructured Interview in the Immunization Clinic

Three parents were recruited (children were interviewed separately) for 90-minute interviews aimed to gain insight into immunization experiences and strategies that helped their child, like “can you tell us what it was like for your child last time you come in for immunizations?” and “what sort of things have you or someone tried to make the experience easier?” Participants were then briefed on VR technology and asked if they thought it could help their child.

#### Semistructured Interviews in the Perioperative Setting

Parents and carers were recruited while in the postoperative waiting area, shortly before discharge from the hospital. Each interview lasted about 10 minutes. We used rating scales and open-ended questions, which were developed by researchers with a background in clinical anesthesia based on issues raised by parents and carers in their clinical practice. Participants rated how much they knew about various aspects of the appointment (4-point scale, ranging from “nothing at all” to “everything”). Open-ended questions included “what were good or difficult aspects of their hospital visit and postoperative time?” and “what can be improved?” and whether they were worried about the operation (yes/no). When children had an experience of prior anesthetics, participants rated the quality of that experience (5-point scale, from “very bad” to “very good”).

## Results

All interviews and focus group recordings were transcribed. Two researchers performed thematic analysis separately and discussed the outcomes until they agreed on a coding scheme based on similarity in meanings associated with participants’ statements, following what Braun and Clarke [19] identify as a bottom-up or inductive approach. The coding scheme included four themes based on the interview data obtained from children and adolescents in the immunization group, which exhibited similarity to the parents’ group in the same setting. The themes were then used as a coding scheme to analyze interview data in all three groups in that setting. Survey data was tabulated in Excel (Microsoft Corporation), and average ratings defined. Participants’ comments in the perioperative setting were analyzed to identify areas of interest and strategies that helped with calming children based on their experiences.

### Clinicians

#### Focus Group

Participants (2 males, 2 females) were anesthetists (n=2), a registered nurse (n=1), and a child life therapist (n=1). Participants worked with children in a variety of settings, including perioperative care, procedural sedation settings, and immunization services. All participants were familiar with VR technology, but none had used it in their practice. Four themes were identified in the participants’ statements (Textbox 1).

**A summary of clinicians’ perspectives on design and intervention strategies that fulfill the experience and psychological needs of pediatric patients to manage their periprocedural anxiety.**

**Experience needs**

- Patient’s prior experience
- Parent’s attitude
- Patient’s attitude

**Psychological needs**

- Sense of agency
- Privacy

**Intervention strategies**

- Set achievable goals for the patient
- Provide distraction
- Improve communication
- Personalize intervention
- Educate

**Design strategies**

- Provide positive emotional experiences
- Embed narrative storytelling
- Accomplishment and satisfaction through game tasks
- Match contextual requirements

#### Experience Needs

The patient’s prior experience and their parent’s attitude towards their child’s anxiety can hinder a child’s ability to manage their periprocedural anxiety or even promote anxiety. In response to prior negative experiences, some children become anxious as soon as the nurse or doctor enters the room. This response is often exacerbated if children detect parental anxiety. Our participants identified three typical personas based on the patient’s attitude: *trusting child* (who is easy to connect with and instruct), *timid child* (engaging with whom requires additional effort by clinicians), and *anxious child* (engaging with whom requires the most effort, and sometimes multiple visits to the hospital).

#### Psychological Needs

Providing a sense of agency (eg, offering realistic choices to the patient) and ensuring privacy throughout the appointment are essential considerations for fulfilling the psychological needs of pediatric patients to manage their anxiety.

#### Intervention Strategies

Typical strategies for pediatric anxiety management include setting achievable goals, so the patient gains a sense of accomplishment, distraction through activities, such as singing songs, blowing bubbles, playing games, communication of information, and allowing time for familiarization. One participant noted, “I gave a mask to a patient to take home, and they practiced diligently every day before the next appointment and were then able to complete the procedure more comfortably.” Participants suggested they tailored their strategies to the specific needs of the patient, such as by adjusting the level or form of distraction. A fundamental difference was evident among the strategies sought by the anesthetists, the child life therapist, and the nurse. Anesthetists’ interactions with children typically last a few minutes. They are focused on negotiating alternative scenarios with the child (eg, a choice between taking the mask or needle injection), whereas the therapist and the nurse in the group wanted to communicate, educate, and prepare children for future appointments.

#### Design Strategies

Participants suggested future VR solutions should provide enjoyable emotional experiences (eg. the thrill of riding a roller coaster), embed narrative storytelling (eg, adventure and exploration), and create a sense of accomplishment in children. Additionally, participants suggested creating virtual experiences that correspond to the events of the physical environments (eg, seeing the wind blow in VR precisely at the time as receiving gas during induction of anesthesia). Physical interaction with the device was noted as important, eg, operating a device with both hands is impractical if one hand must be still for the clinician to establish intravenous access.

#### Survey

Participants were anesthetists (n=23), anesthesia recovery nurses (n=19), and preoperative nurses (n=8). The clinical experience of participants varied from 1 to 30 years (mean 11, SD 8.28). Average clinician ratings indicate their views on what pediatric patients worry about before, during, and after an operation. As shown in Table 1, the top three worries for the 4-7 year age group were: going to sleep with a needle (mean 4.62, SD 0.60), being surrounded by strangers (mean 3.76, SD 0.92), and not knowing what is about to happen (mean 3.68, SD 1.02). In the 8-11 age group, the top three worries were: going to sleep with a needle (mean 4.08, SD 0.72), not knowing what is about to happen (mean 3.76, SD 0.80), and possible pain after the procedure (mean 3.48, SD 0.89). For patients aged 12-17, the top worries were: pain after the procedure (mean 3.94, SD 0.68), waking up during anesthesia (mean 3.92, SD 0.85), and not knowing what is about to happen (mean 3.64, SD 0.98).

When asked what things could make the child feel good, calm, or reassured before an operation, clinicians specified some strategies that were common to all age groups:

- Being friendly and creating a positive experience to set a favorable precedent,
- Creating a calm perioperative environment,
- Using distraction techniques (eg, blowing bubbles) to provide experiences of joy or surprise, was stated as particularly important for younger children (4-7 and 8-11),
- Keeping familiar items such as toys that are comforting to pediatric patients,
- Effective communication and providing adequate, truthful and age-appropriate preoperation information was described as more relevant to older children (12-17),
- Positive and friendly engagement, eg, sitting with the patient rather than standing, particularly with younger age groups (4-7 and 8-11),
- Allowing parents to be present, keeping parents calm, and
- Acknowledging the child’s agency by offering them choices (eg, gas or needle).

**Table 1:** The average rating (likelihood of each item occurring on a 5-point scale ranging from “not at all” to “very much”) of clinician’s responses to the repeated survey on the experience of pediatric patients in three age groups (n=56).

Survey question		Patients		
		4-7 years, mean (SD)	8-11 years, mean (SD)	12-17 years, mean (SD)
**When it comes to the preoperative phase, do you think children are most worried about…**				
	Not knowing what is about to happen?	3.70 (1.02)	3.8 (0.80)	3.6 (0.98)
	Waiting in the pre-op area?	2.6 (0.84)	2.8 (0.97)	2.6 (0.95)
	Going into theatres?	3.6 (0.73)	3.4 (0.25)	3.1 (0.87)
	Being surrounded by strangers?	3.8 (0.92)	2.9 (0.79)	2.2 (0.71)
	Going to sleep with a mask?	3.5 (0.76)	2.9 (0.74)	2.4 (0.86)
	Going to sleep with a needle?	4.6 (0.60)	4.1 (0.72)	3.4 (0.70)
**Reflecting on how kids think about the actual operation, do you think they are worried about…**				
	Complications that might happen during the operation?	2 (0.82)	2.7 (0.92)	3.5 (1.01)
	Waking up during anesthesia?	2.4 (1.14)	3.3 (1.04)	3.9 (0.85)
**Reflecting on what kids think about what will happen after the surgery itself, do you think they are worried about…**				
	Pain after the procedure?	3.2 (0.98)	3.5 (0.89)	3.9 (0.68)
	Feeling sick after the procedure?	2.1 (0.95)	2.6 (0.99)	3.1 (0.97)
	Being okay to go home?	2.5 (1.18)	2.8 (0.91)	3.2 (0.87)
	Complications after the procedure?	1.7 (0.82)	2.3 (0.89)	3.2 (0.90)
	Having a scar after the operation?	1.8 (0.71)	2.6 (1.13)	3.4 (0.92)
	Making a full recovery?	2.1 (1.02)	2.6 (1.16)	3.2 (1.00)

### Children and Adolescents

#### Semistructured Interviews in the Immunization Setting

Three participants (2 males, 1 female) aged 12-15 years (mean 13) were recruited. All participants said they had prior experience of severe anxiety associated with needles. All participants were familiar with VR technology. Four themes were identified (Textbox 2).

**Children and adolescents’ perspectives of their experience and psychological needs and intervention and design strategies that can help them manage their periprocedural anxiety.**

**Experience needs**

- Disconnect from the surroundings through an immersive experience
- Engage in simple activities that are easy to learn
- Engage in recreational activities such as creating art

**Psychological needs**

- Sense of agency
- Experience a range of emotions
- Set emotional goals

**Intervention strategies**

- Provide distraction
- Time the distraction well to start distraction before the procedure begins
- Involve other stakeholders (such as parents) in the intervention
- Build resilience through empathic relationships (eg, with pets)
- Personalize intervention

**Design strategies**

- Embed narrative storytelling
- Familiar yet novel design elements
- Mask unpleasantness, particularly noises

#### Experience Needs

Participants suggested that prior negative experience impedes their ability to manage their anxiety during hospital visits. VR was suggested to be helpful as it is “immersive,” unlike tablet or mobile technology, and it can disconnect them from their physical environment. One participant said, “I just want to get out of there” and wanted to wear the VR headset at the beginning of the appointment to avoid seeing the needle. Simple VR games that are easy to learn and play in a short time were suggested, eg, creating art, playing a favorite sport, singing, and playing music.

#### Psychological Needs

Participants recognized that a useful VR experience must help them calm down autonomously through gameplay. They gave examples of including emotional goals, starting with “excitement” to match their state before receiving an injection (eg, going on exciting VR adventures) and ending with a “relaxing” experience. One participant said, “I know I can never be relaxed immediately, so I want something exciting to begin with.”

#### Intervention Strategies

Distraction strategies were discussed, such as involving other stakeholders in the game experience (eg, an accompanying parent or carer). Participants noted the importance of personalization if these were to occur in VR. One participant wanted to take their pet to the appointment for comfort, which may suggest the relevance of empathic interactions for coping strategies.

#### Design Strategies

Several themed narratives were suggested for a VR app, such as nature, superpowers, learning something new, and playing familiar games. Participants also discussed interactive sensory experiences such as “fast-paced” music to mask procedure noises.

#### Semistructured Interviews in the Perioperative Setting

A total of 65 (44 males, 21 females) participants aged 4-16 (mean 10, SD 3.89) were recruited. Overall, 49 (75%) participants had previous experiences of anesthetics (1 to 17 times, mean 4 times) and rated the quality of those experiences at a mean score of 2, SD 0.81 (4-point scale, “very bad” to “very good”) (Table 2).

Overall, 37 (56%) participants claimed they had worries “about the operation or any other things that might happen before, during, or after the operation.” Worries about what happens while they are asleep were: having bad dreams, inadequate analgesia, duration or depth of sleep, a repeat of a bad experience, and the surgery itself. Worries about the anesthetics were: change of location, loss of sensation, uncontrolled movement while asleep, sharp objects such as needles, and missing friends and school. Finally, worries related to the postoperative experience were: being alone, emotional lability, the presence of an intravenous cannula, overnight admission, removal of tapes, and surgical outcomes.

Concerning children’s knowledge about their hospital visit, the highest rating was given to knowing what happens during the anesthesia (mean 2.28, SD 0.97) and the lowest rating to knowledge about going into the rooms where the operation happens (mean 2.11, SD 0.99).

**Table 2:** Children’s average ratings of how much they knew about the hospital visit on the day of the procedure (4-point scale, ranging from 1=“nothing at all” to 4=“a lot or everything”).

Survey question	Mean (SD)
How much did you know about what it would be like when you got to the hospital?	2.28 (1.02)
How much did you know about where you would spend time before the surgery?	2.19 (1.03)
How much did you know about who you would meet before the operation?	2.19 (0.90)
How much did you know about what it would be like going into the operation room?	2.11 (0.99)
How much do you know about what happens during an anesthetics?	2.28 (0.97)
How much do you know about what happens during surgery?	2.16 (1.01)
How much do you know about what happens to you after the surgery?	2.19 (0.94)

Additional comments revealed opportunities to improve preoperative experiences across all age groups, such as effective communication of preoperative information (knowing they will not be able to feel the pain or needles during the surgery), having a parent present, and anticipation of positive surgical outcomes. Distraction was mentioned by children in age groups 4-7 and 8-11, but not by those age 12+, who additionally mentioned “staff attitude” and “a child-friendly environment.” When asked what could be fun with the anesthetics and operation, responses varied from having access to toys, games, and music, getting well, sleeping, and the hospital facility itself.

### Parents

#### Semistructured Interview in the Immunization Clinic

All three participants identified their child as having severe anxiety related to needles. Only one parent was familiar with VR technology. Details of the four themes are discussed below:

1. *Experience needs*. Participants discussed their emotional experiences, such as helplessness due to their perceived inability to help with their children’s anxiety. One parent felt that they did not have the means or time to prepare their child for appointments at the hospital.
2. *Psychological needs*. Parents mentioned that having information about the procedure affords their child a level of control, whereas feeling that someone is trying to control their child’s response to anxiety only diminishes their ability to manage it. They suggested that being allowed to take personal items to the appointment (eg, their favorite music) is often helpful.
3. *Intervention strategies*. Two strategies were suggested for regulating anxiety during immunization appointments. One was distraction (eg, playing games) so that the child does not “see” the needle, and the other was goal setting to shift their focus from the unpleasant elements.
4. *Design strategies*. Parents described their children as being sensitive (eg, building things up in their head that adds to their anxiety) who sometimes feel guilty for their anxiety. They suggested that a VR experience could help them build a sense of accomplishment and pride.

#### Semistructured Interview in the Perioperative Setting

A total of 35 parents and carers were recruited. Their children were aged 4-16 (mean 9.86, SD 3.85). Overall, 23 (65%) parents identified their children as having prior experiences of anesthesia (prior instances mean 4.3, SD 4.66), with an average quality of prior experiences rated 3.91, SD 1.04 (5-point scale, “very bad” to “very good”). In total, 12 parents were concerned about what might happen before, during, or after the operation.

When asked how they might describe their child at the beginning of anesthesia (4-point scale, “anxious” to “calm”), the average rating was 3.31 (SD 0.76). The average knowledge about various aspects of the procedure is shown in Table 3. Most items were rated above 3, with the lowest ratings given for knowledge about waiting before the operation (mean 3.09, SD 1.03) and what happens during surgery (mean 3.09, SD 0.97).

When asked about what might improve their child’s preparation for anesthesia, parents suggested making children feel safe to set a better precedent for future appointments. The also suggested creating a positive environment (eg, smaller waiting rooms), distracting and keeping the child occupied (eg, gameplay), clear communication of what to expect, positive engagement with trusting and caring staff and allowing a parent to be present at the time of induction.

When asked about what they found difficult at the hospital, participants cited preoperative anxiety and fasting, worrying about the child will wake up during the surgery, the postoperative state of their child (eg, being disoriented), long waiting times, the environment not being child-friendly, and lack of information about the procedure and logistics (eg, car park).

**Table 3:** Parents’ (n=35) average ratings of how much they knew about the procedure before arriving at the hospital (4-point scale, ranging from 1=“nothing at all” to 4=“everything”).

Survey question	Mean (SD)
Before arriving today, how much did you know about what to do when you got to the hospital?	3.32 (0.64)
Before arriving today, how much did you know about what the room where you wait before the operation would be like?	3.09 (1.03)
Before arriving today, how much did you know about who you would meet before the operation?	3.30 (0.92)
Before today, how much did you know about what it would be like going into the actual operating room?	3.18 (0.87)
Before today, how much did you know about what happens during an anesthetic?	3.15 (0.82)
Before today, how much did you know about what happens during surgery?	3.09 (0.97)
Before today, how much did you know about what happens after surgery?	3.21 (0.88)

## Discussion

There is currently a substantial gap in meaningful and truly human-centered applications of VR for managing pediatric anxiety, which can be addressed by putting design and the needs of the patients at the heart of VR development [20]. In order to characterize effective VR design strategies that are suitable for this population, we conducted a multiperspective investigation. Data obtained through a focus group, survey, and interviews allowed us to synthesize the perspectives of three stakeholder groups: patients, parents, and clinicians. Based on the findings, we propose 10 factors that highlight opportunities for future VR design beyond simple distraction, to enhance the experiences of children and adolescents in the periprocedural setting. These are discussed next.

### Factors Determining a Positive Experience for Pediatric Patients

Table 4 summarizes 10 factors to guide the design of future pediatric VR solutions.

**Table 4:** A list of 10 design factors that can be used as input when devising a solution to assist children with managing their periprocedural anxiety. Groups of participants (clinicians, children, and parents) who have mentioned each factor are identified with an “x.”

Design factors	Example	Clinicians	Children	Parents
Empathic experience	Diversifying a range of positive experiences such as reassurance, empathy, calm	x	x	x
Welcoming Environment	Creating a child-friendly and warm environment with minimal complexity in procedures	x	x	x
Stimulation & distraction	Offering fun and enjoyable experiences (eg, positive surprise) that distract from negative stimuli	x	x	x
Personalized strategies	Setting achievable goals that are tailored to the child’s ability and providing a sense of accomplishment	x	x	x
Effective communication	Providing useful information and answering questions helps children deal with their anxiety and increases their confidence	x	x	x
Engagement with staff	Positive engagements with staff improve emotional support and reduce anxiety	x	x	x
Involving parents	Feeling that parents are close and part of the procedure provides a feeling of safety and diminishes anxiety in children	x	x	x
Acknowledging agency	Feeling in control and having some choices provides a sense of agency and confidence In managing anxiety	x	x	x
Fulfilling emotional needs	Acknowledging the child’s emotional expectations helps them cope with their anxiety (eg, not expecting them to calm down immediately and helping them to work through their emotions)		x	x
Familiar design	Familiar forms of technology and design can improve the child’s belief that they have the tools to manage their anxiety	x	x	x

#### Experience

VR can offer a positive experience of reassurance, empathy, and calm to negate children’s anxiety. Empathy, in particular, is demonstrated valuable in *Farmoo*, a VR app that allows pediatric cancer patients to care for a virtual farm and companion character [21].

#### Welcoming Environment

Immersive VR environments can establish a sense of presence in a child-friendly, intimate, and age-appropriate virtual space with proportionality cultivating confidence and preventing the child from feeling small.

#### Stimulation and Distraction

Our findings suggest distraction paired with stimulating emotional experiences such as positive surprise, is desirable and may help children disconnect from distressing elements of the environment. Positive emotions are shown to alleviate psychological stress [22] and improve the efficacy of distraction through VR [18].

#### Personalized Strategies

Distraction in VR can be tailored to a patient’s age and abilities and preferences to set achievable goals and elicit a sense of accomplishment, as noted by parents and clinicians in our study. All three stakeholder groups agreed that VR solutions should affirm the child’s belief that they have the tools to manage their anxiety. From our findings, we can infer that the clinicians’ perception of “what would make a child feel good, calm and reassured” is primarily based on their existing knowledge of an average child’s cognitive abilities appropriate to that age group. Therefore, for the oldest age group (12-17), clinicians cited “effective communication such as providing adequate, truthful and age-appropriate preoperative information” while considering “using distraction techniques” and “positive staff engagement” as vital to the 8-11 and 4-7 age groups. However, children from all three age groups cited that it was effective communication that made them feel good about going to sleep for the operation. Although distraction was mentioned by the younger children (4-7 and 8-11), it was not mentioned by those in the adolescent group. This result was different from the clinicians’ response that distraction was a priority for the 12-17 age group.

#### Effective Communication

Our findings demonstrated the importance of providing adequate information for reducing pediatric anxiety. In our study, 56% (n=36) of children and nearly 30% (n=12) of parents in the preoperative setting expressed worry about all stages of an operation when their knowledge about the operating room was lowest. This result confirms the need for better communication of information with patients and their carers. There have been some examples in this area, with mixed results. Ryu et al [7] showed a VR tour of the operating room to children aged 4-10 and found it significantly reduced their anxiety. However, Liszio and Masuch’s [23] playful VR simulation of an MRI procedure for children aged 8-15 did not produce a significant reduction in patients’ anxiety. Instead, it reduced the parents’ anxiety and enhanced clinician satisfaction. Eijlers et al [24] similarly used VR to show the operating room to children (n=191, aged 2-12) before surgeries. They also found no significant changes in self-reported anxiety; however, patients needed rescue analgesia significantly (*P*=.002) less often (55%) than the control group (95.7%). The challenges of measuring the efficacy of VR intervention are not trivial. Although self-reported anxiety was not reduced in these cases, meaningful clinical measures (ie, rescue analgesic requirement) did confirm a significant difference. Future studies may need to examine the impact of integrating multiple design factors (in Table 4) into a holistic VR plan for children to manage their anxiety.

#### Engagement With Staff

In addition to supporting the implementation of VR solutions, staff can partake in a virtual experience to further connect with the patient and even personalize the VR app based on the patient’s age and abilities. *Voxel Bay,* for example, is a VR app where some game features are controlled by the clinician to provide a positive surprise to the child [8].

#### Involving Parents

Similar to engagement with staff, VR provides an excellent platform to create a parental presence during the procedure. All three groups of stakeholders in our study stated this is crucial for managing pediatric patient’s anxiety.

#### Acknowledging Agency

All groups involved in our study confirmed that fostering agency is vital to pediatric patient’s ability to manage their anxiety. Agency is exemplified in *Voxel Bay* VR app [8] in the form of several choices afforded to the patient from customizing their cardboard VR headset to picking a companion game character.

#### Fulfilling Emotional Needs

Our research identified emotional goal-setting as a unique strategy to support anxiety management. Children interviewed in our study independently identified that at the commencement of procedures, significant anxiety results in a state of heightened arousal, which a VR experience should match before any attempt to induce relaxation or distraction. To our knowledge, this remarkable finding has not been implemented in any study or existing app. Surprisingly, this strategy was not suggested by clinician participants in our research, further illustrating the relevance of a multiperspective approach for mapping future design opportunities.

#### Familiar Design

Both clinicians and children in our study stated that having familiar items at the hospital is comforting. This need for a comfort item can be achieved in VR by embedding a familiar design or music in the app, potentially reducing barriers to app learnability, as noted by our young participants.

### Future Design Directions for Pediatric VR Solutions

**Figure 1:**
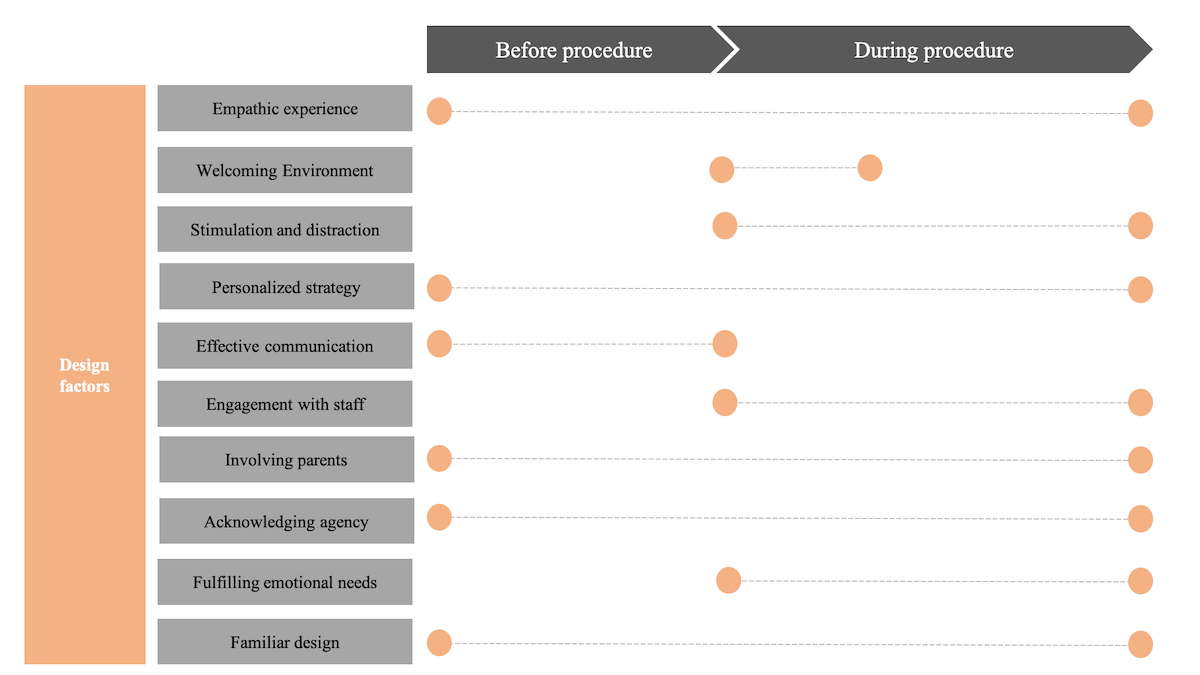
The patient journey mapped across the medical procedure phases (before and during) with design factors that can make an impact in either or both of those phases.

The diversity of the 10 design factors generated based on our findings reveals the complexities and potential challenges in meeting the experiential, psychological, and intervention needs of pediatric patients and their carers through one VR solution. Ahmadpour et al [18] called for skill-building in VR to allow patients to become active agents in their care in a variety of ways. Building meaningful skills in VR necessitates a holistic approach due to the temporality of periprocedural experience with different needs becoming prominent at different stages of the procedure. Therefore, any individual VR solution cannot encompass all 10 factors. Instead, the factors can be mapped to address specific needs at different points of the patient’s journey (before the medical procedure and during the procedure), as shown in Figure 1.

This effort may start with a *communication-focused* VR app to familiarize the patient and carers with the details of their procedure in advance. This can then be coupled with an *procedure-focused* VR solution to strategically shift the patient’s focus and set emotional goals to help them manage their anxiety autonomously. Design elements that are stimulating, welcoming, and engaging of staff or parents through gameplay, may enhance the effectiveness of these apps.

The research presented in this paper is part of the larger Kids Immersive VR (KiDiVR) project, a collaboration between The University of Sydney and The Children’s Hospital at Westmead, which aims to establish a suite of VR apps to augment the pediatric patient journey. This study helped us scope and define the opportunities within the KiDiVR suite. The next phase will involve ideation and prototyping. Specifically, we seek to create a novel VR app to test the efficacy of using emotional goals to guide patient arousal from high to low in order to reduce their anxiety. Our approach is iterative, and therefore we will seek feedback from multiple stakeholders throughout our process to ensure their values are captured, and their needs are met.

### Limitations

This study has several limitations. We collected qualitative and quantitative data using tailored questions. This decision was made due to the exploratory nature of our study as a preliminary effort to capture the values and needs of multiple stakeholder groups. The findings should, therefore, be verified using validated tools in the future. The validity of the 10 proposed design factors should be examined using VR prototypes and through controlled clinical trials. We identified a limitation in methods used to assess the efficacy of pediatric VR apps in the literature when testing mainly relied on self-reported anxiety. Future research could address this limitation by investigating the efficacy of VR apps using a combination of meaningful clinical measures (eg, need for rescue analgesia), physiological measures of anxiety, and qualitative assessment (eg, satisfaction), as suggested by Ahmadpour et al [18].

Our participants were recruited from one hospital in Australia. The sample size in our immunization group was small, which limited our interactions with this potential but important target user group. Although we were able to recruit a wide range of respondents in our perioperative group, more diversity in the sample and recruitment from other institutions may result in different perspectives. There is also the potential for selection bias to be introduced, as volunteers for this form of research may represent a more engaged and active group than the general population. All clinicians in the focus group were familiar with VR technology, as were the children and adolescents in the immunization group. All but one parent from the latter group were also familiar with VR. The clinicians and participants in the immunization group were recruited based on direct contact, which may be classified as a convenience sampling approach. Our participants in the immunization group self-identified as having severe anxiety, which was not formally assessed. These may be considered as limitations of our study and challenge the reliability of our findings. We will address these limitations in future studies by recruiting a larger sample using random methods to ensure participants represent a variety of subgroups (eg, those not familiar with VR technology) and performing data triangulation.

### Conclusions

This research highlights the value of obtaining multiple perspectives for identifying the needs and values of patients. We identified 10 factors that may inform future VR solutions to enrich the periprocedural experiences of children and adolescents. A striking similarity was evident in the key factors identified by children, parents, and clinicians, including personalization of strategies to help children build skills to deal with their periprocedural anxiety. Many existing VR apps are used to mediate distraction techniques equally to all users irrespective of their abilities, needs, and preferences. There is an opportunity to maximize the utility of VR as a procedural support tool at different points of patients journey, particularly by using a communication-focused solution before the appointment and an procedure-focused solution during the appointment to set meaningful goals for the patient.

## Abbreviations

HMD: head-mounted display
HCI: human-computer interaction
KiDiVR: Kids Immersive VR
MRI: magnetic resonance imaging
VR: virtual reality
